# Acetylation Regulates WRN Catalytic Activities and Affects Base Excision DNA Repair

**DOI:** 10.1371/journal.pone.0001918

**Published:** 2008-04-09

**Authors:** Meltem Muftuoglu, Rika Kusumoto, Elzbieta Speina, Gad Beck, Wen-Hsing Cheng, Vilhelm A. Bohr

**Affiliations:** 1 Laboratory of Molecular Gerontology, National Institute on Aging, National Institutes of Health, Baltimore, Maryland, United States of America; 2 Institute of Biochemistry and Biophysics, Polish Academy of Sciences, Warszawa, Poland; University of Minnesota, United States of America

## Abstract

**Background:**

The Werner protein (WRN), defective in the premature aging disorder Werner syndrome, participates in a number of DNA metabolic processes, and we have been interested in the possible regulation of its function in DNA repair by post-translational modifications. Acetylation mediated by histone acetyltransferases is of key interest because of its potential importance in aging, DNA repair and transcription.

**Methodology/Principal Findings:**

Here, we have investigated the p300 acetylation mediated changes on the function of WRN in base excision DNA repair (BER). We show that acetylation of WRN increases in cells treated with methyl methanesulfonate (MMS), suggesting that acetylation of WRN may play a role in response to DNA damage. This hypothesis is consistent with our findings that acetylation of WRN stimulates its catalytic activities *in vitro* and *in vivo*, and that acetylated WRN enhances pol β-mediated strand displacement DNA synthesis more than unacetylated WRN. Furthermore, we show that cellular exposure to the histone deacetylase inhibitor sodium butyrate stimulates long patch BER in wild type cells but not in WRN depleted cells, suggesting that acetylated WRN participates significantly in this process.

**Conclusion/Significance:**

Collectively, these results provide the first evidence for a specific role of p300 mediated WRN acetylation in regulating its function during BER.

## Introduction

In eukaryotes, DNA is packaged into highly structured chromatin filaments, which are further compacted into chromosomes. The structure of eukaryotic chromatin and chromosomes influences accessibility of proteins and multi-protein machines to eukaryotic DNA, thereby influencing the efficiency and timing of DNA metabolic processes such as transcription, replication, DNA repair and recombination. Post-translational modification of chromatin proteins, including histones, plays a crucial role in regulating these metabolic processes [1], [2]. For example, acetylation of N-terminal lysine residues generates a more open and accessible chromatin configuration [3]–[5] and is associated with increased gene expression. Histone acetylation is catalyzed by histone acetyltransferases (HATs) such as p300/CBP and PCAF. p300 also acetylates several DNA repair proteins [6]–[9] and transcriptional regulators [10]. Acetylation of histones and other non-histone proteins is reversed by histone deacetylases (HDACs); thus, HATs and HDACs coordinately regulate acetylation status [11]. Hyperacetylation of histones H3 and H4 enhances DNA excision repair in UV-irradiated cells, suggesting that the extent of chromatin acetylation modulates DNA repair [12]–[15]. The class III HDAC protein, sir2, prolongs life span in yeast by locking chromatin into a silent state [16]. Recently, Mostoslavsky et al. [17] suggested that inactivation of another sirtuins, SIRT6, causes genome instability and premature aging in mice by inhibiting base excision repair (BER).

Werner syndrome (WS) is an autosomal recessive segmental progeria caused by mutations in the gene encoding Werner protein (WRN). WS patients prematurely develop atrophic skin, thin gray hair, osteoporosis, type II diabetes, cataracts, arteriosclerosis, and cancer [18], [19], symptoms that are highly reminiscent of normal aging. WRN belongs to the RecQ family of DNA helicases and has 3′-5′ helicase and exonuclease activities. WRN plays a role in maintaining genomic stability, and may play a role in DNA repair [20]. Several studies correlate WS with a defect in repair of oxidative DNA damage [21]–[25], which may directly contribute to the phenotype of premature aging observed in WS patients. This idea is consistent with the fact that WRN stimulates polymerase β (pol β)-mediated DNA synthesis [22], [26], an essential step in BER. However, the exact physiological role of WRN in DNA metabolism, including BER, is still unclear.

A key element in understanding the function of WRN in DNA repair may be its post-translational modifications. We have previously observed that WRN catalytic activities are severely modified by its phosphorylation status [27], [28]. Both serine/threonine and tyrosine phosphorylation of WRN inhibits WRN helicase and exonuclease activities, and may stimulate translocation of WRN from the nucleolus to sites of DNA damage [27], [28]. Acetylation of WRN by p300 also stimulates translocation of WRN from the nucleolus to the nucleoplasm [29]. However, the consequences of altered subcellular localization, and the functional effects of phosphorylation or acetylation of WRN are not yet known.

Recent studies have demonstrated that p300 acetylates BER proteins including pol β, 8-oxoguanine-DNA glycosylase 1 (OGG1), and NEIL2 (a glycosylase for oxidized pyrimidines) [6], [7], [9], and that acetylation of these proteins affects their functions differentially. For example, acetylation of pol β specifically inhibits its dRP lyase activity, but has no effect on its polymerase or AP-lyase activities [9]. In addition, acetylation by p300 inhibits the glycosylase activity of human NEIL2 [6] while it stimulates the glycosylase activity of OGG1, suggesting that OGG1 acetylation might be important for removal/repair of 8-oxoG and other damaged bases [7]. Despite much work in this area, the extent to which acetylation regulates BER is still poorly understood.

In this study, we have examined the role of p300 acetylation in regulating WRN functions in the context of BER. The results show that acetylation of WRN by p300 stimulates WRN ATPase, helicase and exonuclease activities *in vitro* and *in vivo*. The level of WRN acetylation increases in cells exposed to methyl methanesulfonate (MMS), and long-patch BER activity increases in wild type but not in WRN knockdown cells treated with sodium butyrate. Acetylation of WRN also stimulates pol β-mediated strand displacement DNA synthesis. These results are consistent with the hypotheses that acetylation regulates an early step in response to DNA damage for which, WRN may be an important immediate downstream target of p300, and that acetylation of WRN contributes to the regulation of BER.

## Results

### Acetylation of WRN *in vivo* is stimulated by MMS

**Figure 1:**
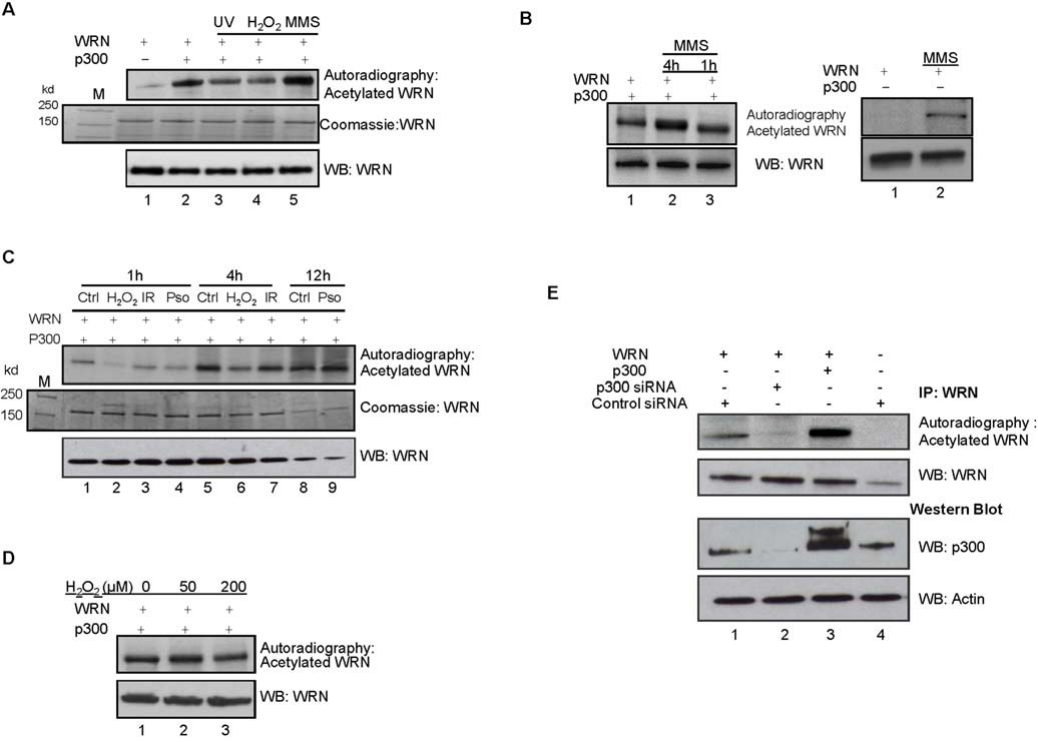
WRN is acetylated *in vivo* and it is stimulated by MMS treatment. (A) WRN was transiently overexpressed in 293T cells either alone or together with p300. 48 h after transfection, untreated, UV, H_2_O_2_ or MMS treated cells were labeled with 1 mCi/ml [^3^H]sodium acetate for 1 h. [^3^H]acetate labeled WRN was immunoprecipitated using an anti-WRN antibody and the complex was resolved on SDS-PAGE and was analyzed either by autoradiography (upper panel) or Western analysis (lower panel) or coomassie-stained gel after the acetylation assay, which was subsequently, analyzed using autoradiography (middle panel). (B) Right and left top panels: 48 h after transfection, the cells were treated with 1 mM MMS and WRN acetylation was followed for 1 h and 4 h (left panel) or 1 h (right panel), and labeled with 0.5 mCi/ml [^3^H]sodium acetate for 1 h. [^3^H]acetate labeled WRN was immunoprecipitated using an anti-WRN antibody and the complex was resolved on SDS-PAGE and analyzed by autoradiography. Lower panels: Western analysis of acetylated samples with an anti-WRN antibody. (C) Top panel: 48 h after transfection, the cells were treated with H_2_O_2_ (1 and 4 h), γ-irradiation (1 and 4 h) or psoralen+UVA (1 and 16 h) and labeled with 0.5 mCi/ml [^3^H]sodium acetate for 1 h. [^3^H]acetate labeled WRN was immunoprecipitated using an anti-WRN antibody and the complex was resolved on SDS-PAGE and analyzed by autoradiography. Medium panel: the coomassie staining of the gel before autoradiography. Lower panel: Western analysis of acetylated samples with an anti-WRN antibody. (D) Top panel: After transfection, the cells were treated with 50 µM and 200 µM H_2_O_2_ and labeled with 0.5 mCi/ml [^3^H]sodium acetate for 1 h. Lower panel: Western analysis of acetylated samples with an anti-WRN antibody. (E) 293T cells were transiently transfected with expression vectors of WRN (lanes 1–3), p300 (lane 3), siRNA of p300 (lane 2) or siRNA negative control (lanes 1 and 4) individually or in combination. [^3^H]acetate labeled WRN was immunoprecipitated using an anti-WRN antibody and the complex was resolved on SDS-PAGE and was analyzed either by autoradiography (first panel) or Western analysis (second panel). Two bottom panels are Western analysis of these cell lysates with anti-p300 and anti-β-Actin antibodies, which shows relative protein expression levels.

To determine the physiological conditions under which WRN are acetylated in cells, we transiently transfected 293T cells with WRN expression plasmid alone or together with p300 expression plasmid. Acetylated proteins were radiolabeled *in vivo* by incubating the cells with 1 mCi/ml [^3^H]sodium acetate (Fig. 1A, upper panel) or 0.5 mCi/ml [^3^H]sodium acetate (Figs. 1B–E) under conditions with or without treatments with 20 J/m^2^ UV, 250 µM H_2_O_2_, 1 mM MMS, γ-irradiation (6 Gy) or 0.1 µg/ml psoralen+UVA. The acetylation status of WRN was then analyzed by autoradiography after immunoprecipitations of WRN with the anti-WRN antibody. As shown in Fig. 1A (upper panel), lane 1, the immunoprecipitated WRN was specifically labeled in the presence of [^3^H]sodium acetate, implying that it is acetylated *in vivo*. Co-transfection of WRN with p300 revealed a 6-fold increase in WRN acetylation (compare lanes 1 and 2) indicating that p300 contributes to WRN acetylation *in vivo*. In addition, the accumulation of acetylated WRN was found to be time-dependent. As shown in Fig. 1C, upper panel, lane 1, acetylation of WRN was detected at 1 h after addition of [^3^H]sodium acetate and the level of [^3^H]acetate labeled WRN had increased significantly at 4 h (Fig. 1C, upper panel, lane 5).

We found that MMS treatment of the cells for 1 h increased the acetylation of WRN approximately 2-fold compared to untreated cells (Fig. 1A, upper panel, compare lanes 2 and 5, and Fig. 1B, left panel, compare lanes 1 and 3). Moreover, 1 mM MMS treatment further increased the acetylation of WRN after 4 h compared to MMS treatment at 1 h and untreated cells (Fig. 1B, left panel, compare lanes 2 and 3 and lanes 1 and 2, respectively). This effect was not due to an increase in the amount of immunoprecipitated WRN, because the coomassie staining of the gel before autoradiography and Western analysis of acetylated samples with anti-WRN antibody indicated that the same amount of WRN was immunoprecipitated (Fig. 1A, medium and lower panels, compare lanes 2 and 5, and Fig. 1B, left panel, lanes 1–3). We also showed that MMS enhances the acetylation of WRN in the cells expressing endogenous p300 only (Fig. 1B, right panel). Treatment of the cells with UV (Fig. 1A, upper panel, lane 3), H_2_O_2_ (Fig. 1A, upper panel, lane 4 and Fig. 1C, upper panel, lanes 2 and 6), γ-irradiation (Fig. 1C, upper panel, lanes 3 and 7) and psoralen+UVA (Fig. 1C, upper panel, lanes 4 and 9) did not increase p300 acetylation of WRN. On the basis of coomassie staining of the gel before autoradiography (Fig. 1C, medium panel) and Western blot analysis of the same acetylated samples (lower panel), the protein levels of WRN were quite similar among the loaded samples. The incubation of the cells with 250 µM H_2_O_2_ for 1 h, and associated acetylation of WRN for 1 h and 4 hrs, showed that under these conditions WRN acetylation decreased (Fig. 1A, upper panel, lane 4 and Fig. 1C, upper panel, lanes 2 and 6). To rule out the possibility that we were detecting a phenomenon caused by cell toxicity or overdose, we conducted an experiment lowering H_2_O_2_ concentration to 50 µM. While 200 µM H_2_O_2_ caused a decrease in the WRN acetylation (Fig. 1D, upper panel, lane 3), we found that using 50 µM H_2_O_2_ the acetylation status of WRN did not change (Fig. 1D, upper panel, compare lanes 1 and 2). Fig. 1D, lower panel, shows the Western analysis of the same acetylated samples. Taken together, these results indicate that WRN is acetylated *in vivo* by p300 and that the acetylation is most prominent after MMS treatment.

To assess whether p300-induced WRN acetylation is directly mediated by p300, we suppressed p300 expression by p300 siRNA knockdown in 293T cells transfected with a WRN expression vector, and measured WRN acetylation with autoradiography as described above. As shown in Fig. 1E (upper panel), over-expression of p300 led to augmented acetylation of WRN (compare lanes 1 and 3), whereas suppression of endogenous p300 siRNA reduced WRN acetylation (compare lane 2 with lanes 1 and 3). WRN acetylation was detected in cells transfected with WRN expression vector but not in mock-transfected cells (compare lanes 1, 3 and 4). Fig. 1E, panels 2 and 3, shows relative levels of WRN and p300 protein expression. Western analysis of these cell lysates showed the reduced expression of endogenous p300 by p300 siRNA in 293T cells (Fig. 1E, panel 3, compare lane 2 with 1 and 4). Actin was used as a loading control (Fig. 1E, bottom panel). These results confirm that WRN acetylation *in vivo* is mediated by p300.

### WRN is acetylated *in vitro* by p300 and the acetylation sites are located at the N- and C-terminal domains

**Figure 2:**
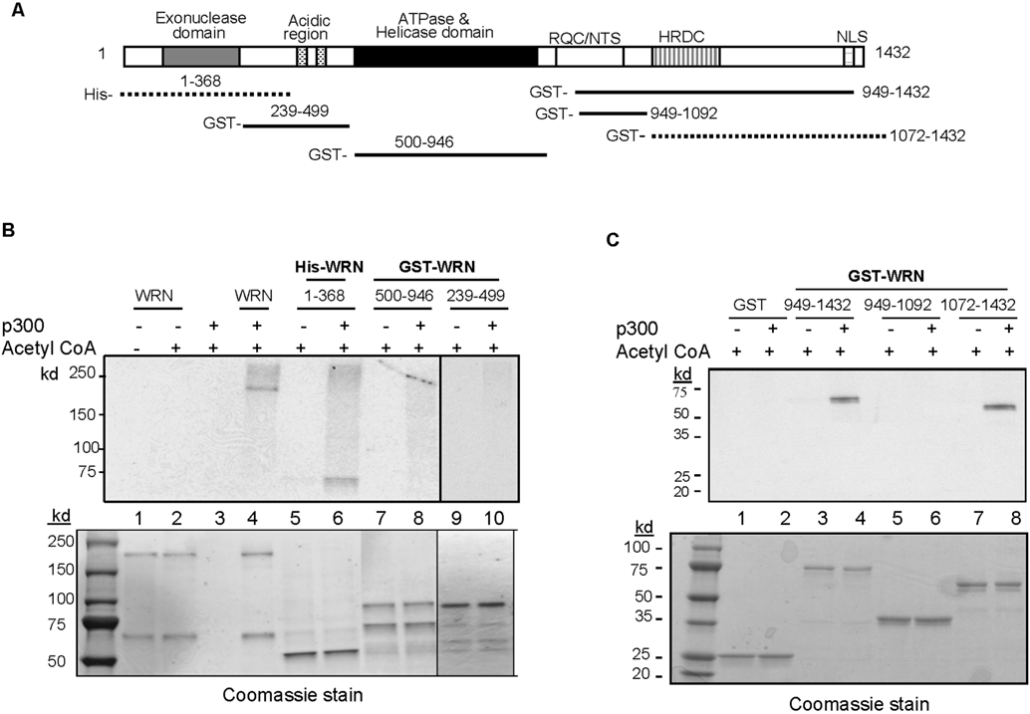
Mapping of the WRN acetylation site. (A) Schematic representation of functional domains of WRN and GST-tagged WRN fragments. RQC, RecQ-conserved domain; NTS, nucleolar targeting sequence; HRDC, helicase and RNase D conserved domain; NLS, nuclear localization signal. Numbers indicate WRN amino acid sequence. (B) One microgram of WRN and WRN fragments were incubated with [^14^C]-acetyl-CoA in the presence or absence of p300. The lower panel shows a coomassie-stained SDS-PAGE gel of purified WRN and GST-WRN fragments after the acetylation assay, which was subsequently, analyzed using autoradiography (top panel). (C) One microgram of GST (control) and GST-WRN fragments were incubated with [^14^C]-acetyl-CoA in the presence or absence of p300. The lower panel shows a coomassie-stained SDS-PAGE gel purified GST-WRN fragments after the acetylation assay that was analyzed using autoradiography (top panel).

We examined *in vitro* acetylation of WRN by incubating 1 µg of WRN with [^14^C]acetyl CoA and 100 ng of recombinant human p300 for 60 min at 30°C. The reaction products were run on a SDS-PAGE gel and [^14^C]-acetate incorporation of WRN was analyzed by autoradiography. As shown in Fig. 2B, WRN was labeled by [^14^C]acetyl CoA in the presence of p300 *in vitro* (Fig. 2B, upper panel, lane 4). This *in vitro* acetylation was not due to WRN autoacetylation or nonspecific interaction between WRN and [^14^C]acetyl CoA (Fig. 2B, upper panel, lanes 1–3).

Using a series of recombinant truncated WRN variants (Fig. 2A), we mapped the p300-dependent acetylation sites of WRN *in vitro* to the N-terminal (aa 1–368) (Fig. 2B, upper panel, lane 6) and C-terminal (aa 949–1432) domains of WRN (Fig. 2C, upper panel, lane 4). For fine mapping of the WRN acetylation sites, the C-terminal domain was further sub-fractioned into two regions including the RQC (aa 949–1092) or the HRDC and C-terminal NLS domains (aa 1072–1432) (Fig. 2A). The WRN acetylation site enacted by p300 resided within a region of WRN that harbors the HRDC and C-terminal NLS domains (aa 1072–1432) (Fig. 2C, upper panel, lane 8). The WRN fragments together with acetyl CoA were used for *in vitro* acetylation to determine if there were nonspecific interactions between acetyl CoA and these fragments or if there was autoacetylation of the fragments (Fig. 2B, upper panel, lanes 5, 7 and 9; Fig. 2C, upper panel, lanes 3, 5, and 7). Since the WRN fragments were GST-tagged (Fig. 2A), GST was used in the acetylation reaction and as shown in Fig. 2C, lane 2 (upper panel), GST was not acetylated by p300. Figs. 2B and 2C (lower panels) show a coomassie-blue stained SDS-PAGE gel of the purified WRN and GST-WRN fragments after the acetylation assay, which was subsequently analyzed using autoradiography (upper panel).

### Acetylation of WRN increases its catalytic activities

**Figure 3:**
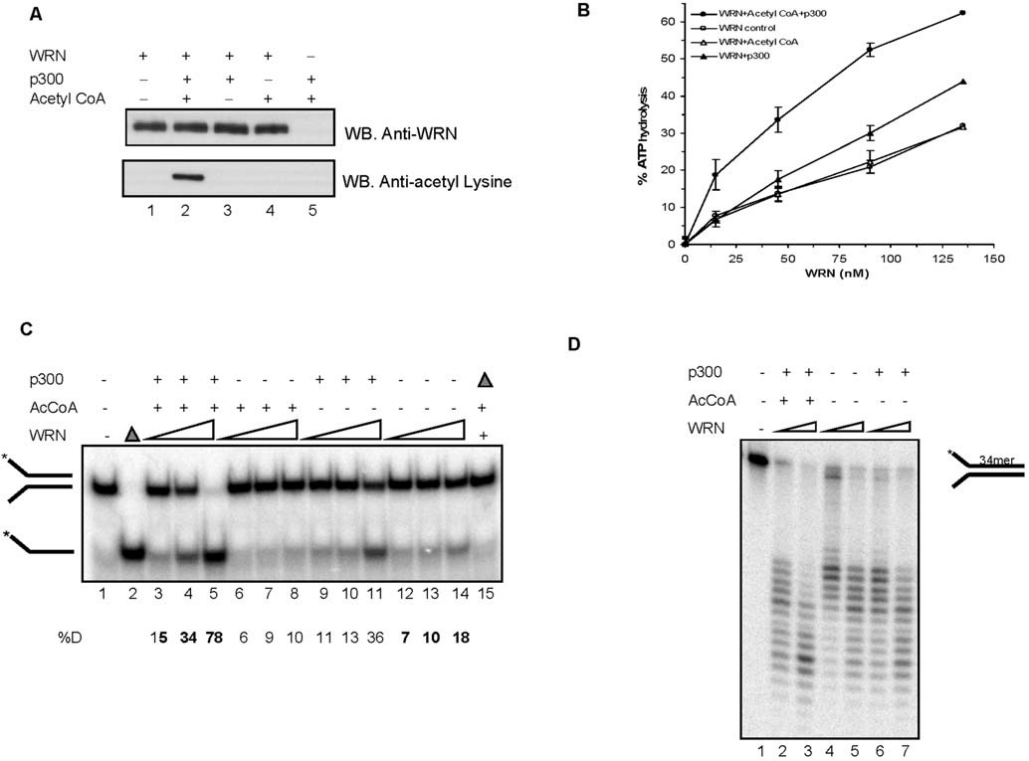
Effect of acetylation on WRN catalytic activities. (A) One microgram of WRN was incubated with in the presence or absence of acetyl CoA or p300 or both. The top panel shows a WB of WRN after the acetylation assay, which was stripped and analyzed using anti-acetyl lysine antibody (bottom panel). These reaction mixtures were used in the measurement of WRN ATPase (B), helicase (C) and exonuclease activities (D). (B) Acetylation increases the ATPase activity of WRN. [^32^P]ATP and 150 ng of M13mp18 ssDNA was incubated with increasing concentrations of nonacetylated (open circles) and acetylated WRN (closed circles) (15, 45, 90, 135 nM). WRN was incubated with p300 (closed triangles) or with acetyl CoA (open triangles). ATP hydrolysis was analyzed on PEI-TLC plates developed in 1 M formic acid/0.8 M LiCl. Plates were scanned using a PhosphorImager. The percentage of ATP hydrolysis was determined by each sample using the ImageQuant software and subtracting background value in control having no enzyme. (C) The effect of acetylation on WRN helicase activity. Reactions containing increasing amounts (0.125 nM, 0.25 nM and 0.5 nM) of nonacetylated WRN (lanes 12 to 14) and acetylated WRN (lanes 3 to 5) were incubated with a 22 bp forked substrate (0.5 nM, 22Fork3/22Fork4) for 15 min at 37°C. Lanes 6 to 8, WRN in the presence of acetyl CoA. Lanes 9 to 11, WRN in the presence of p300. Lane 1, substrate only. Lane 2, heat-denatured substrate. Reaction products were run on a 12% native gel and visualized using a PhosphorImager. %D, percentage of single-stranded product displaced. (D) The effect of WRN acetylation on WRN exonuclease activity. Reactions contained 10 and 20 nM nonacetylated WRN (lanes 4–5) and acetylated WRN (lanes 2–3), and WRN plus p300 (lanes 6–7) were incubated with a 34 bp forked substrate (0.5 nM, 34ForkA/34ForkB) for 15 min at 37°C. Lane 1, no enzyme. Products were heat-denatured for 5 min at 95°C, run on a 14% denaturing polyacrylamide gel and visualized using a PhosphorImager.

To gain further insight into the functional significance of p300-mediated acetylation of WRN, we investigated whether acetylation affects the catalytic activities of WRN. Purified WRN was acetylated *in vitro* by p300 either in the presence (Fig. 3A, lower panel, lane 2) or absence of acetyl CoA (lane 3), acetyl CoA alone, or WRN alone (lane 4 or 1) for 60 min at 30°C as described above. The reaction products were analyzed by Western analysis using an anti-WRN antibody. The results showed that there were equal amounts of WRN in each reaction (Fig. 3A, upper panel, lanes 1–5). Analysis of the same blot by anti-acetyl-lysine antibody showed that WRN was acetylated only in the reaction containing both p300 and acetyl CoA (Fig. 3A, lower panel, lane 2). These reaction mixtures were then used to measure WRN ATPase, helicase, exonuclease and DNA binding activities.

First, we examined the effect of acetylation on the ATPase activity of WRN. WRN DNA-dependent ATPase activity was measured by incubating increasing concentrations of acetylated or unacetylated WRN (15, 45, 90 and 135 nM) with circular M13mp18 ssDNA and γ-[^32^P]ATP. The fraction of hydrolyzed ATP increased with increasing unacetylated WRN (Fig. 3B, open circles). However, the rate of ATP hydrolysis was significantly higher (2.5 fold) at all WRN concentrations when the reaction was performed using acetylated WRN (Fig. 3B, closed circles). Although acetyl CoA alone did not stimulate WRN ATPase activity (Fig. 3B, open triangles), the presence of p300 weakly stimulated WRN ATPase activity even in the absence of acetyl CoA (Fig. 3B, closed triangles). These results show that p300 acetylation of WRN increases its ATPase activity.

Next, we used a 22 bp forked duplex substrate (Fig. 3C) [30] to determine whether acetylation of WRN affects its helicase activity. WRN helicase activity increased with increasing concentration of unacetylated WRN (Fig. 3C, lanes 12–14) or acetylated WRN (Fig. 3C, lanes 3–5), but was significantly higher in the presence of acetylated WRN. At the highest concentration of WRN, acetylation stimulated WRN helicase activity approximately 4.3-fold (Fig. 3C, lanes 5 and 14). Although acetyl CoA alone did not stimulate WRN helicase activity (Fig. 3C, lanes 6–8), p300 stimulated WRN helicase activity by 2-fold at the highest WRN concentration even in the absence of acetyl CoA (Fig. 3C, lanes 11 and 14). The ability of p300 to stimulate WRN helicase activity was heat labile (lane 15), suggesting that it is likely intrinsic to the p300 protein. Furthermore, p300 alone did not unwind the substrate (data not shown), indicated that p300 does not have an inherent helicase activity nor does the p300 protein preparation contain contaminating DNA unwinding activity. Acetylation by p300 also stimulated WRN exonuclease activity significantly (Fig. 3D, lanes 2–5); p300 also stimulated WRN exonuclease, but the stimulation was significantly higher in the presence of acetyl CoA than in the absence of acetyl CoA (Fig. 3D, lanes 6–7). These results demonstrate that acetylation of WRN by p300 strongly stimulates WRN catalytic activities, while p300 alone moderately stimulates WRN catalytic activities, independent of its ability to acetylate WRN protein.

### Acetylation enhances the exonuclease activity of WRN *in vivo*

**Figure 4:**
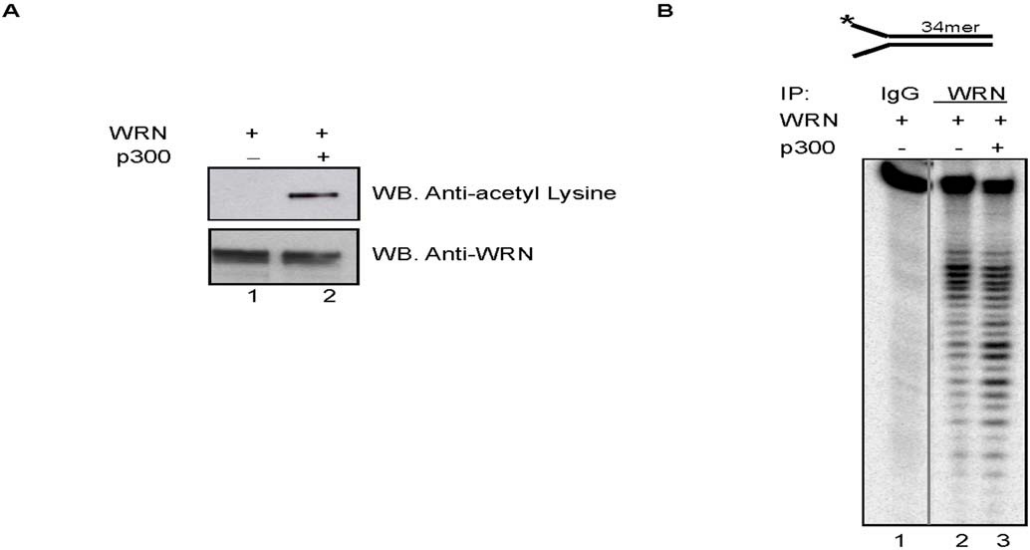
Acetylation of WRN increases its exonuclease activity *in vivo*. (A) The top panel shows a WB of 293T cell extract transfected with or without p300 plasmid analyzed using an anti-acetyl lysine antibody; bottom panel shows the same membrane stripped and analyzed with an anti-WRN antibody. These cell extracts were used in the measurement of WRN exonuclease activities (B). (B) Lysates from 293T cells with (lane 3) or without (lanes 1 and 2) transfection with p300 expression plasmid were immunoprecipitated with antibodies against immunoglobulin G (IgG) (lane 1) or WRN (lanes 2 and 3). The washed immunocomplexes were incubated with the exonuclease substrate (top, 0.5 nM, final concentration) as described.

To investigate the potential roles for acetylated WRN in cells, we measured exonuclease activity in WRN immunocomplexes from 293T cells transfected with WRN expression plasmid alone or together with the p300 expression plasmid. The washed anti-IgG or anti-WRN immuno-precipitates were incubated with the exonuclease substrate. Fig. 4B shows that the anti-WRN immunoprecipitates digested the 34-bp forked duplex substrate, and that the digestion was enhanced after the transfection of cells with p300 (Fig. 4B), which caused the acetylation of WRN (Fig. 4A). These results suggest that WRN acetylation increased the exonuclease activity in the WRN complex *in vivo*.

### WRN forms a complex with p300 and p300 interacts with the acidic domain of WRN (WRN_239–499_)

**Figure 5:**
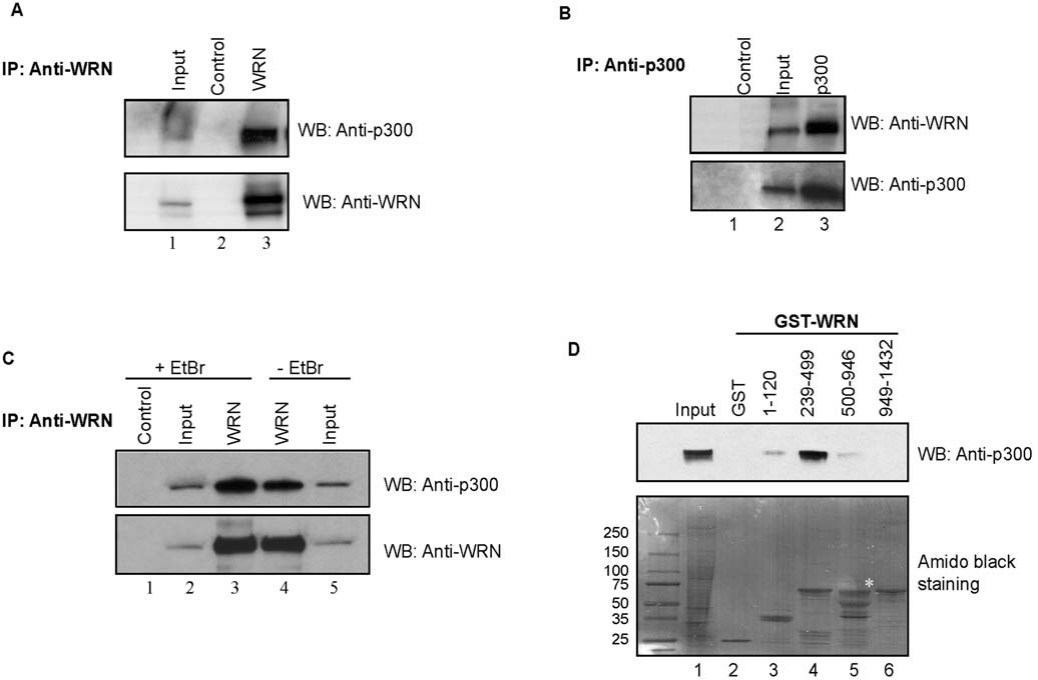
Characterization of the WRN/p300 physical interaction. (A) Co-immunoprecipitation of WRN and p300. HeLa nuclear extracts were immunoprecipitated with either polyclonal rabbit anti-WRN antibodies (lane 3) or control IgG (lane 2). The immunoprecipitates were analyzed by SDS-PAGE and Western analysis with anti-p300 or anti-WRN antibodies, as indicated. Input, 10% loaded (lane 1). (B) HeLa nuclear extracts were immunoprecipitated with either polyclonal rabbit anti-p300 antibodies (lane 3) or control IgG (lane 1). The immunoprecipitates were probed with anti-WRN or anti-p300 antibodies, as indicated. Input, 10% loaded (lane 2). (C) WRN and p300 were co-immunoprecipitated from HeLa nuclear extracts either in the absence (lanes 4 and 5) or the presence (lanes 1 to 3) of ethidium bromide (Et.Br.; 50 µg/ml) with anti-WRN antibodies (lanes 2 and 5) but not with the control antibodies (lane 1), as indicated by Western analysis with anti-p300 antibodies (upper) and anti-WRN antibodies (lower). Inputs are 10% (lanes 2 and 5). (D) HeLa nuclear extracts (400 µL) were incubated with either GST alone (lane 2) or GST-tagged WRN fragments (lanes 3–6) that were prebound to glutathione beads. Eluted proteins were separated by SDS-PAGE, transferred to a membrane, and stained with amido black to ensure equal loading of the various WRN fragments. The membrane was probed with mouse anti-p300 antibody. The input (lane 1) corresponds to 5% of HeLa nuclear extracts used in the binding reactions. The *asterisk* indicates mobility of full-length GST-WRN helicase domain (aa 500–946).

To examine whether p300 is in complex with WRN *in vivo*, we performed co-immuno-precipitation experiments using HeLa nuclear extracts, which were pre-treated with the endonuclease DNase I. The incubation of the extracts with an antibody against WRN specifically immunoprecipitated endogenous WRN as expected (Fig. 5A, bottom panel, lane 3), and also co-precipitated endogenous p300 (Fig. 5A, upper panel, lane 3). This result was confirmed by a reciprocal co-immunoprecipitation experiment, in which antibody to p300 co-immuno-precipitated endogenous p300 and WRN (Fig. 5B, upper panel, lane 3). Control experiments showed that pre-immune IgG did not immunoprecipitate p300 or WRN (Fig. 5A, upper panel, lane 2 and Fig. 5B, upper panel, lane 1). These results demonstrate that WRN forms a complex with p300 *in vivo*. DNase I degrades exposed DNA, potentially breaking the tether between two DNA-bound proteins, but does not intercalate and unwinds the DNA. To ascertain that the interaction between WRN and p300 was not mediated by DNA, WRN and p300 were co-immunoprecipitated from HeLa nuclear extracts pre-treated with or without ethidium bromide (EtBr), which disrupts non-specific DNA−protein interactions by intercalating and unwinding the DNA helix (Fig. 5C). The presence of EtBr did not disrupt the co-precipitation of WRN and p300 (Fig. 5C, compare lanes 3 and 4). Instead, disruption of the DNA binding increased the immunoprecipitation of WRN (Fig. 5C, bottom panel, lane 3) and consequently its co-immunoprecipitation with p300 (upper panel, lane 3). This may be due to the release of WRN or p300 from DNA, allowing them to associate more readily. Since WRN forms a complex with p300 in the presence of EtBr (Fig. 5C, upper panel, lane2), it can be concluded that this protein-protein interaction is not mediated by DNA. Furthermore, ELISA experiments also suggest that WRN and p300 interact directly (Fig. S1).

The region of WRN that interacts physically with p300 was mapped using GST-tagged fragments of recombinant WRN and analyzing which of these fragments retain the ability to bind p300. GST fusion proteins were bound to glutathione beads, and mixed with HeLa nuclear extracts in the presence of DNase I. The bound protein fraction was eluted and analyzed by Western analysis. The results suggest that the acidic domain contained in aa 239–499 of WRN plays an essential role in binding p300 (Fig. 5D, upper panel, lane 4), because WRN fragments lacking this region failed to bind to p300 in this assay (lanes 3, 5 and 6). Control experiments showed that p300 was not precipitated by GST (lane 2) and WRN GST-fusion proteins were present in similar amounts during the binding assay (Fig. 5D, lower panel). These results suggest that p300 specifically interacts with WRN through its acidic domain.

**Figure 6:**
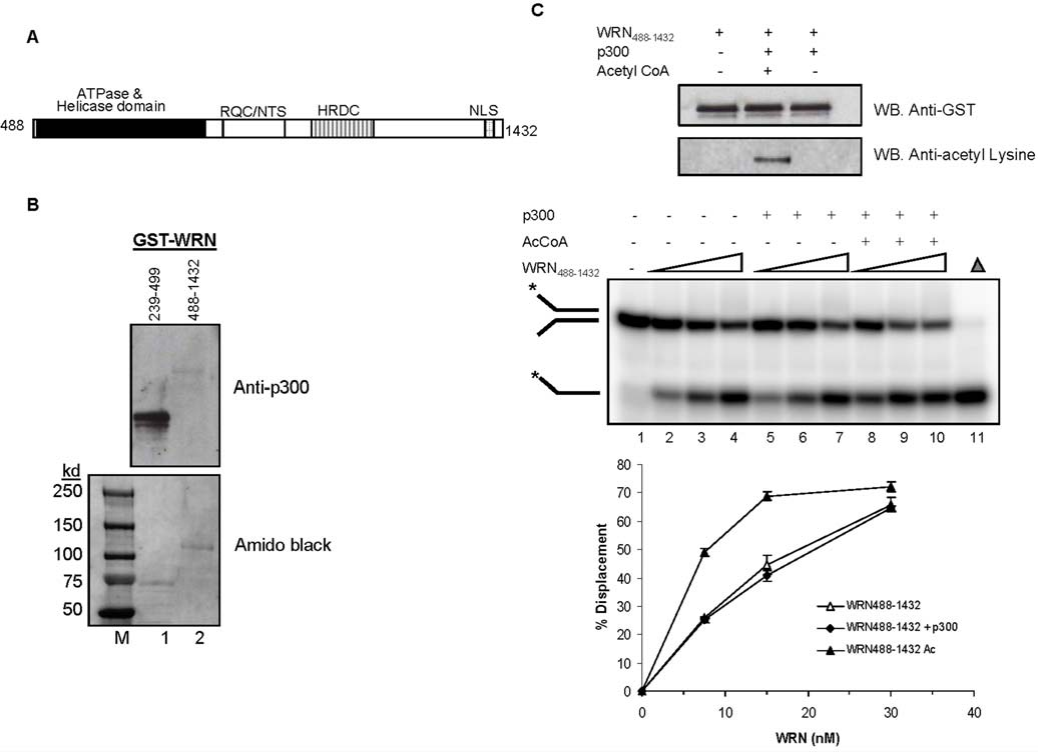
Acetylation of WRN_488–1432_ stimulates its helicase activity. (A) A schematic representation of GST- WRN_488–1432_ fragment. (B) Recombinant WRN_239–499_ and WRN_488–1432_ fragments were separated by SDS-PAGE, transferred to a membrane, and stained with amido black to ensure equal loading of the WRN fragments (bottom panel, lanes 1 and 2). The membrane was blotted with p300 protein and probed with mouse anti-p300 antibody (top panel, lanes 1 and 2). (C) One microgram of WRN_488–1432_ fragment was incubated with in the presence or absence of acetyl CoA or p300 or both. The top panel shows Western analysis results of WRN_488–1432_ fragment after the acetylation assay, which was stripped and analyzed using an anti-acetyl lysine antibody (bottom panel). These reaction mixtures were used in the measurement of helicase activity (medium panel). Medium panel: Reactions containing increasing amounts (7.5, 15, and 30 fmol) of nonacetylated WRN_488–1432_ fragment (lanes 2 to 4) and acetylated WRN_488–1432_ fragment (lanes 8 to 10) were incubated with a 22 bp forked substrate (0.5 nM, 22Fork3/22Fork4) for 15 min at 37°C. Lanes 5 to 7, WRN_488–1432_ fragment in the presence of p300. Lane 1, substrate only. Lane 11, heat-denatured substrate. Reaction products were run on a 12% native gel and visualized using a PhosphorImager. Bottom panel: Quantitation of percentage of unwinding product.

To distinguish the effect of direct interaction between p300 and WRN and that of acetylated WRN on its catalytic function, we constructed a GST tagged-WRN fragment lacking the p300-binding domain (WRN_488–1432_) (Fig. 6A). Far-Western analysis was used to compare binding of WRN_488–1432_ and WRN_239–499_ to p300. The results demonstrated that p300 does not bind to WRN_488–1432_ (Fig. 6B, upper panel, lane 2), but it does bind to WRN_239–499_ (Fig. 6B, upper panel, lane 1). Because WRN_488–1432_ has helicase activity but not p300 binding activity (Fig. 6C, middle panel, lanes 2–4), it was possible to test the effect of p300 acetylation on WRN catalytic activity in the absence of p300 binding. For this experiment, purified recombinant WRN_488–1432_ was acetylated by p300 in the presence or absence of acetyl CoA for 60 min at 30°C as described previously. The reaction products were analyzed by Western analysis using an anti-GST antibody and an anti-acetyl lysine antibody to confirm the presence of equal amounts of WRN_488–1432_ and the acetylation status of WRN in each reaction, respectively (Fig. 6C, upper panel). Helicase assays showed that p300 alone did not stimulate the helicase activity of WRN_488–1432_ (Fig. 6C, middle panel, lanes 5–7). In contrast, acetylation of WRN_488–1432_ by p300 did stimulate WRN helicase activity (middle panel, lanes 8–10). Quantitative analysis of the helicase activity of acetylated and unacetylated WRN_488–1432_ confirmed this conclusion (Fig. 6C, lower panel). These data demonstrate that p300 has two separate effects on WRN function: p300 stimulates WRN catalytic functions by binding to the transcriptional coactivator domain, but also stimulates WRN by acetylating the WRN_488–1432_ fragment. Thus, p300 appears to play a significant role in regulating WRN catalytic activity.

### Acetylation stimulates WRN DNA binding

**Figure 7:**
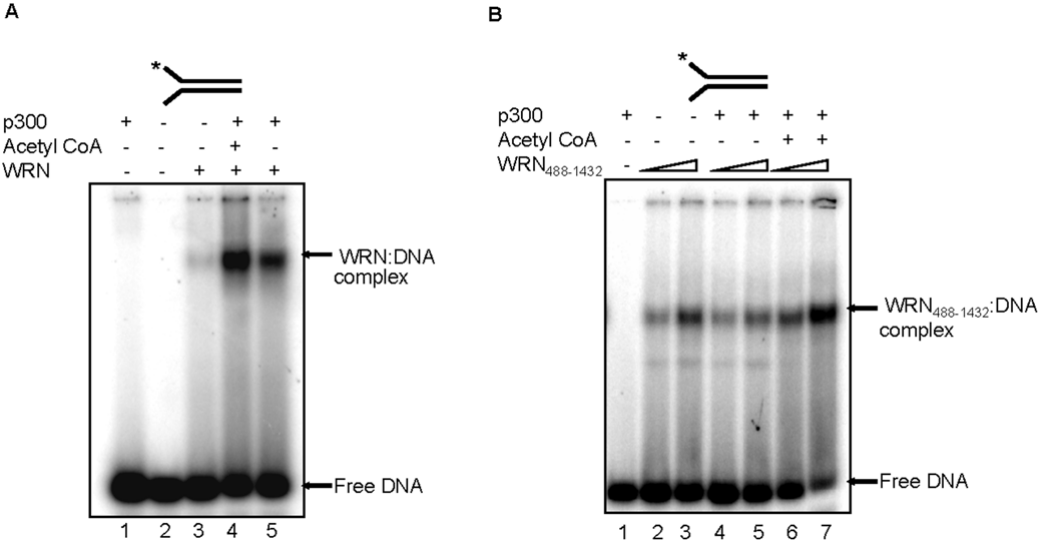
Acetylation of WRN and WRN_488–1432_ stimulates their DNA binding activities. Reactions were run under the conditions described in Materials and Methods on a 5% acrylamide gel and visualized using a PhosphorImager. (A) Acetylated WRN and WRN-p300 complex have higher DNA binding affinity. (B) Acetylated WRN_488–1432_ has higher DNA binding activity.

Because the mechanism by which acetylation of WRN stimulates its catalytic activities is unknown, we examined the effect of acetylation on WRN DNA binding activity using a forked-duplex substrate [30] and a gel-shift assay. The results showed that the DNA binding activity of acetylated WRN (Fig. 7A, lanes 3 and 4) is greater than for p300 alone, without acetylation (Fig. 7A, lane 5). Furthermore, p300 alone did not stimulate DNA binding by WRN_488–1432_, but acetylation of WRN_488–1432_ stimulated its DNA binding activity (Fig. 7B). These results are consistent with previous results shown above, indicating that acetylation stimulates the catalytic activities of WRN and WRN_488–1432_, while p300 only stimulates the catalytic activities of WRN (Figs. 3 and 6). These data also suggest that the functional effects of p300 acetylation or p300 binding on WRN are mechanistically related to increased WRN DNA binding activity.

### Acetylation regulates the role of WRN in BER

**Figure 8:**
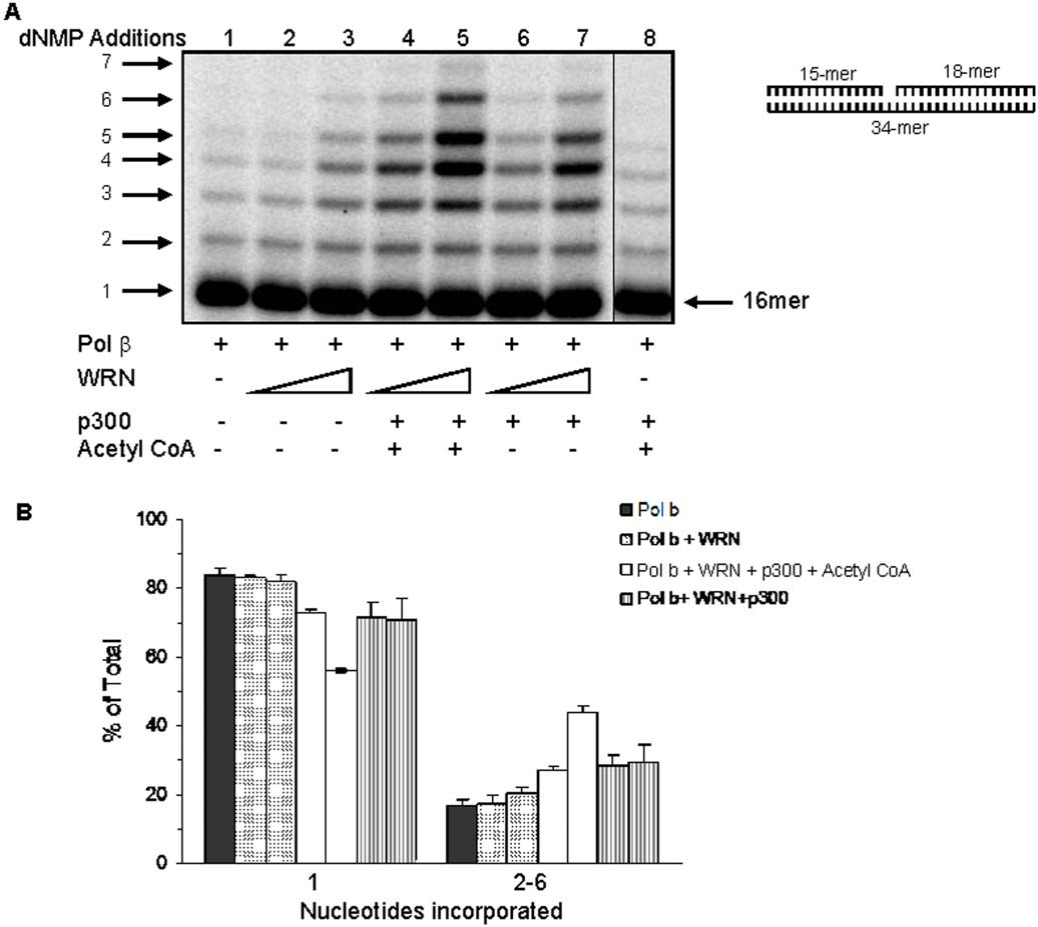
Acetylation of WRN stimulates strand displacement DNA synthesis by Polβ. (A) Reactions contained Pol β alone (1 nM) or together with increasing concentrations of WRN (6 and 12 nM) or acetylated WRN or WRN plus p300 or p300 plus acetyl CoA. The reactions were initiated by adding 12.5 nM one nucleotide gap substrate and were incubated for 25 minutes at 37°C, followed by analysis on a 20% denaturing gel. (B) Quantitation of short (1 nt) and long-patch (2–6) BER intermediates.

Recent studies show that WRN knockdown cells are hypersensitive to MMS and have reduced short and long patch (LP) BER activities [22]. We also demonstrated that WRN stimulates pol β strand displacement DNA synthesis and LP BER [26] and that acetylation of WRN increases after MMS treatment (Fig. 1). These results suggest that acetylation may regulate the function of WRN in BER processes in response to methylation-induced DNA damage. To explore this possibility, we first analyzed the effect of acetylated WRN on pol β strand displacement DNA synthesis (Fig. 8). We reconstituted a BER reaction with a 34-bp substrate containing a single nucleotide gap at position 16. Without addition of DNA ligase, 16-mer oligonucleotide products represent incorporation of a single [α-^32^P]dCMP nucleotide and short patch BER (Fig. 8A, lane 1). The dRP-lyase activity of pol β was inactive on the gapped substrate used in our reconstituted strand displacement assay, allowing us to examine the effect of WRN acetylation on pol β DNA synthesis without interfering with the previously reported inhibition of dRP-lyase activity of pol β after p300 acetylation [9]. In agreement with previous results [22], WRN stimulates pol β-catalyzed strand displacement synthesis in a concentration-dependent manner (Fig. 8A, lanes 2–3). The results also show that acetylation of WRN specifically stimulates production of LP BER intermediates (2–7 nt added) (Fig. 8A, lanes 2–5). In particular, the fraction of short patch BER intermediates (1 nt added) was approximately 1.5-fold lower, while the fraction of LP BER intermediates (2–7 nt added) was approximately 2.2-fold higher in the presence of acetylated WRN than in the presence of unacetylated WRN (Fig. 8B shows quantification of reactions with 1 nM pol β and 12 nM WRN). Control reactions indicate that p300 and acetyl CoA had no effect on the strand displacement DNA synthesis of pol β (Fig. 8A, lane 8). These results suggest that acetylated WRN stimulates strand displacement synthesis by pol β more strongly than unacetylated WRN. We also observed that WRN and p300 stimulate strand displacement DNA synthesis by pol β in the absence of acetyl CoA (Fig. 8A, lanes 6–7 and 8B). However, the effect of p300 in the absence of acetyl CoA was approximately 2-fold lower than its effect in the presence of acetyl CoA.

**Figure 9:**
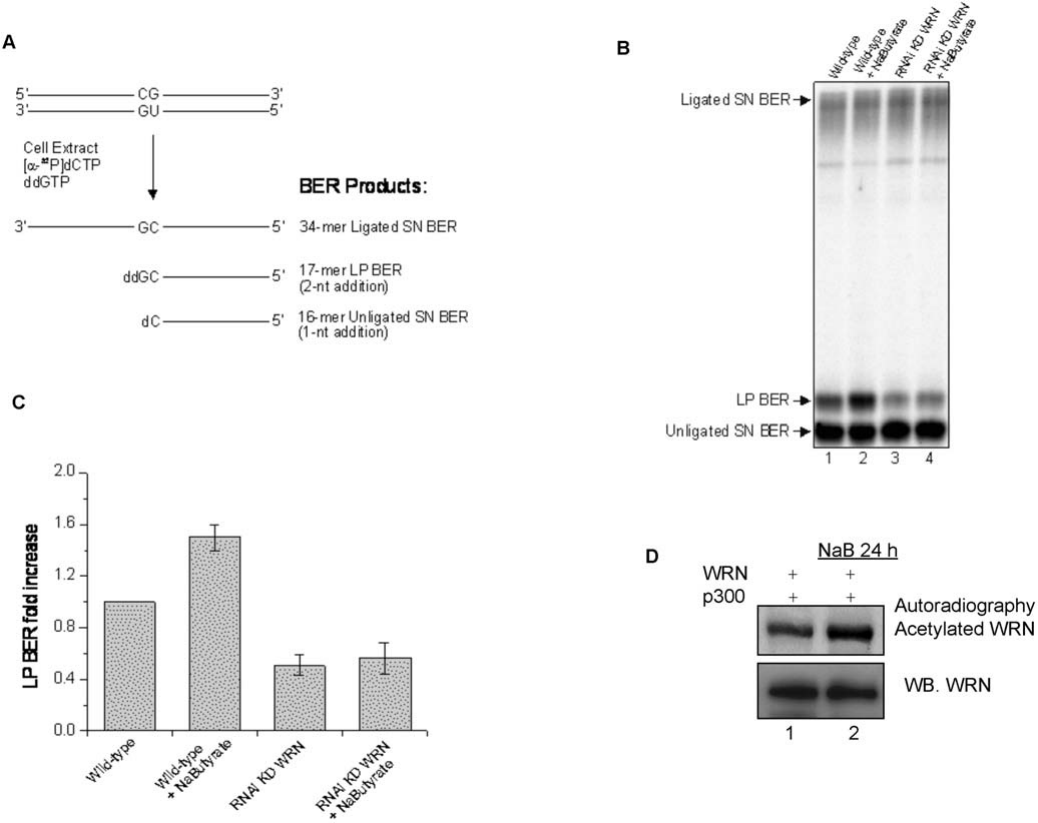
Sodium butyrate treatment enhances LP BER. Comparison of uracil-DNA mediated BER in extracts from wild-type (WT) and WRN KD cells treated with or without 5 mM sodium butyrate. (A) Substrate DNA and predicted BER reaction products and intermediates. The sizes of intermediates (LP BER, 2 nt addition; SN or LP BER, 1 nt addition) and complete BER (ligated 34mer SN BER) product are shown. (B) Phosphorimages of denaturing polyacrylamide gels showing *in vitro* BER products. The reaction conditions and products analyses are described in Materials and Methods. A 34 bp duplex substrate containing a uracil residue at position 16 was incubated with [^32^P]dCTP and ddGTP and WT or WRN KD cell extracts (with or without treatment with NaB), as indicated. The positions of the BER products are indicated. (C) Quantitation of LP BER products from three different experiments is plotted. (D) Top panel: After transfection, the cells were treated with 5 mM sodium butyrate for 24 h and labeled with 0.5 mCi/ml [^3^H]sodium acetate for 1 h. [^3^H]acetate labeled WRN was immunoprecipitated using an anti-WRN antibody and the complex was resolved on SDS-PAGE and analyzed by autoradiography. Lower panel: Western analysis of acetylated samples with anti-WRN antibody.

To further examine the contribution of acetylated WRN or that of acetylation in general to BER, we used a BER assay that specifically examines pol β-mediated LP BER activity *in vivo* [22]. In this assay, a stable WRN shRNA knockdown (WRN KD) and wild type cell lines (shRNA negative control), previously generated and characterized in our lab were used [22]. The WRN KD cells have no detectable WRN expression [22]. Extracts from these cells were incubated with a 34-bp duplex substrate containing a single uracil residue at position 16 in a reaction containing [^32^P]dCTP and chain terminator ddGTP (Fig. 9A) to measure pol β specific incorporation. In this assay, the first nucleotide incorporated by pol β is [^32^P]dCTP, which radiolabels the short patch BER reaction product (Fig. 9B). When ddGTP is inserted, it terminates DNA synthesis; thus the second nucleotide incorporated (17mer) with ddGTP represents a LP BER product (Fig. 9B). DNA ligase, which is present in the cell extracts, ligated some of the radiolabeled oligonucleotides, but no reaction products containing ddGTP could be ligated. Thus, the 34-mer ligated products are fully repaired products of SP BER (Fig. 9B). Quantification of the results of this assay (Fig. 9C) indicates that 25% of total BER was LP BER in wild type cells (Fig. 9B, lane 1). In WRN KD cells, LP BER products decreased approximately 2-fold (Fig. 9B, lanes 1 and 3), indicating that WRN stimulates pol β-mediated LP BER.

We next treated the cells with the histone deacetylase inhibitor sodium butyrate (5 mM) for 24 h and analyzed the BER products as described above (Fig. 9B). Sodium butyrate increases acetylation of both histone and non-histone proteins [31], [32]. In this assay we used a 34-bp oligonucleotide duplex as a substrate (Fig. 9A), and thus we examined the results of sodium butyrate-induced acetylation of BER proteins rather than any influences on chromosomal arrangements. Sodium butyrate treatment increased the percent of LP BER synthesis of total BER by ∼ 2-fold (Fig. 9B, compare lanes 1 and 2). Quantitation of the 17-mer LP BER product without including SP and ligated products showed that sodium butyrate enhances the proportion of 17-mer LP BER products by 1.63-fold (Fig. 9C). A concentration-dependent BER assay using extracts from wild-type cells with or without sodium butyrate treatment revealed that sodium butyrate increased the LP BER intermediates at all concentrations examined (Fig. S2A). These results suggest that sodium butyrate-induced protein acetylation increases the pol β-mediated LP BER pathway.

WRN would be one of the acetylated proteins contributing to the sodium butyrate stimulated LP BER since sodium butyrate was found to increase p300 acetylation of WRN (Fig. 9D, compare lanes 1 and 2). To test the extent of WRN involvement in BER, we measured LP BER in sodium butyrate treated cells lacking the WRN protein. In contrast to the situation in the wild type cells, LP BER (17-mer) was not stimulated in WRN KD cells after sodium butyrate treatment (Fig. 9B, compare lanes 3 and 4; Fig. 9C). The quantitation of LP BER products in WRN KD cell extracts is shown in Fig. 9C and demonstrates that sodium butyrate treatment did not cause any significant increase in LP BER products. We observed the same results with different cell extract concentrations (Fig. S2B). We also explored the role of the acetylated WRN in LP BER by using WRN proficient and the WRN KD cells after MMS treatment, since this treatment increases the acetylation of WRN (Fig. 1). The results demonstrated that the enhancement of pol β strand displacement DNA synthesis after MMS treatment was higher in WRN proficient cells than inWRN KD cells (Fig. S3A and B). These results collectively demonstrate that cellular acetylation is associated with an increase in LP BER, and this increase requires the presence of WRN protein.

## Discussion

It is proposed that WRN contributes to the maintenance of genomic stability through its involvement in DNA repair pathways [33]. WS cells are hypersensitive to various types of DNA damaging agents, including H_2_O_2_, MMS, γ-irradiation and psoralen+UVA [22]–[25], [34]. Post-translational modifications of WRN could be an important mechanism for the regulation of WRN functions in response to DNA damage. Acetylation is one of the most important post-translational modifications for efficient DNA repair. Histone acetylation regulates chromatin assembly facilitating access of DNA repair proteins to the sites of DNA lesions [4]. Histone acetyltransferase, p300, can also acetylate and interact with several DNA repair proteins; the events modulate their activity, protein interactions and cellular localization [6]–[9], [35]. This study provides the first evidence that p300 acetylation of WRN stimulates its catalytic activities *in vitro* and *in vivo* and may play a key role in regulating its function in LP BER. Here, a mechanistic basis for the effect of acetylation on WRN function was characterized in detail. This study confirms an important role for WRN in BER, and a specific role for p300 acetylation in regulating WRN function during the course of BER.

It was previously shown that WRN participates in the repair of MMS induced DNA base damage via both short patch and LP BER [22], [26], [36]. The present study demonstrates that alkylation damage strongly stimulates acetylation of WRN (Fig. 1), and that acetylation of WRN by p300 stimulates its catalytic activities (Figs. 3, 4 and 6) that stimulate pol β-mediated strand displacement DNA synthesis and LP BER (Fig. 8 and 9). Furthermore, treatment of wild type and WRN KD cells with sodium butyrate stimulates LP BER in wild type cells but not in WRN KD cells (Fig. 9). These data strongly support the ideas that p300 acetylation of WRN is an important step in regulating BER in cells with DNA damage.

WRN plays a role in BER during the repair of certain lesions [22], [26], [36]. It can unwind several BER intermediates such as single-strand break intermediates, and stimulate pol β strand displacement DNA synthesis via its helicase activity [26], [36]. Since pol β does not have an intrinsic editing function, it makes frequent errors during nucleotide incorporation [37]. The exonuclease domain of WRN has proofreading activity that can remove 3′ mismatches following misincorporation of nucleotides by pol β, and thus potentially enhance the fidelity of BER [38]–[40]. It is likely that WRN exonuclease and helicase activities facilitate pol β mediated strand displacement and accuracy of BER [22]. Strong acetylation of WRN after alkylation damage might enhance the fidelity of DNA repair synthesis.

*In vitro* studies with reconstituted nucleosome core particles suggest that highly condensed chromatin inhibits some steps in BER [41], [42]. In particular, uracil DNA glycosylase (UDG) and AP endonuclease (Ape1) activities are approximately 10-fold lower in chromatin than in “naked” DNA, and pol β-catalyzed DNA synthesis is completely inhibited by nucleosomes *in vitro* [41], but DNA ligase I and Fen-1 are not inhibited by nucleosomes *in vitro* [43], [44]. These data suggest that pol β-catalyzed DNA synthesis may be the rate-limiting step in BER *in vivo*, and that the major point of restriction of BER in chromatin is the synthesis step of pol β [41]. This indicates that increased acetylation might facilitate recovery from alkylation DNA damage by two distinct complementing mechanisms [4], [45], [46]. Firstly, acetylation of histones might promote chromatin decondensation. Secondly, acetylation of BER proteins, including WRN, might specifically stimulate LP BER. The results presented here support the latter possibility, demonstrating that WRN acetylation stimulates pol β-mediated strand displacement DNA synthesis and LP BER (Figs. 8 and 9). It is also possible that acetylated WRN could be more efficient to unwind DNA at or near sites of DNA lesions. We also show that the interaction between p300 and WRN enhances its DNA binding. Since p300 cannot bind DNA itself [4], WRN is likely to recruit p300 or other histone acetyltransferases such as PCAF to damaged sites, promoting chromatin remodeling. Supporting this hypothesis, Sharma et al. showed that WRN helicase is required for the stable recruitment of PCAF (p300/CBP-binding protein)/P-TEFb-containing transcription complexes to DNA [47]. Thus, while acetylation of histone and non-histone proteins may play a general and global role in the response to DNA damage, and the activity of other BER proteins may be downregulated or upregulated by p300 acetylation [6]–[9], it is proposed here that WRN is a key immediate downstream target of p300 acetylation, especially in cells with alkylation DNA damage. Nevertheless, additional experiments are needed to determine the relative importance of acetylation of other BER proteins by p300 on the efficiency of BER *in vivo*. In addition, the possibility that other acetyltransferase activities in addition to p300 modulate BER *in vivo* cannot be ruled out at this time.

Once BER is completed, WRN might be downregulated by histone deacetylases (HDACs), which might also help reestablish nucleosome positioning, chromatin condensation and epigenetic programming. There are three classes of HDACs in humans: class I and II HDACs, and the silent information regulator 2 (sir2) family (HDAC class III) [48]. Sirtuins are nicotinamide adenine dinucleotide (NAD^+^) dependent and are inhibited by nicotinamide [48], [49], while trichostatin A (TSA) and sodium butyrate inhibit class I and II HDACs. Interestingly, TSA and sodium butyrate stimulate acetylation of WRN, suggesting that class I and/or class II HDACs deacetylate WRN *in vivo*. It needs to be evaluated whether sirtuins deacetylate WRN and work together with class I or II HDACs. It has been reported that TSA stimulated translocation of WRN from the nucleolus into the nucleoplasm [29].

Previous studies examined the effect of sodium butyrate on nucleotide excision repair (NER) [12]–[15] and double strand break (DSB) repair [50], [51]. These studies demonstrated that sodium butyrate stimulates NER in UV-irradiated cells by inducing chromatin hyperacetylation [12]–[15] and that sodium butyrate inhibits repair of DSBs in human cancer cells by inhibiting expression of proteins required for DSB repair including Ku70, Ku86 and DNA-PKcs [50]. Our study is the first examination of the effect of sodium butyrate on the cellular BER process showing that sodium butyrate-induced acetylation stimulates LP BER in wild type but not in cells lacking WRN. This result is consistent with *in vitro* results showing that p300 acetylation of WRN enhances pol β strand displacement DNA synthesis, and suggests that acetylation of WRN by p300 plays a role in regulating BER, as discussed above. The increase in LP BER after sodium butyrate is not due to change in the pol β protein level (data not shown) or to acetylation of pol β. Cells might have evolved a protective mechanism to maintain WRN activity without affecting the amount of the protein after acetylation, hereby increasing the processivity of pol β in BER. Although it seems likely that the effect of sodium butyrate on LP BER is mediated at least in part by increased acetylation of WRN, it remains possible that the effect of sodium butyrate is mediated by modulating the interaction between WRN and other proteins involved in BER.

In conclusion, we propose that acetylated WRN may be important for both the reorganization of chromatin structure and for BER. WRN may recruit p300 to the damaged chromatin site thereby supporting p300 to remodel chromatin during the BER process. After WRN has completed its function in BER, it might be deacetylated by class I or II HDACs, and this would facilitate the reorganization of the chromatin structure. Thus, the genome would remain fully functional for other metabolic processes. Because HDACs such as Sir2 have been shown to increase life span in certain organisms [52] and sodium butyrate and TSA induce cellular senescence, acetylation status of WRN may also have implications for cellular lifespan and senescence. It will be of interest to explore this possibility in future studies.

## Materials and Methods

### Recombinant proteins

Recombinant histidine-tagged human WRN [53], histidine-tagged N-WRN (amino acids 1–368), Glutathione S Transferase (GST)-WRN fragments [54] were purified as described previously.

### *In vivo* acetylation assay

To detect WRN acetylation *in vivo*, 293T cells were transfected with WRN plasmid (3 µg) either alone or in combination with a p300 expression plasmid (7 µg). 48 h after transfection, the cells were treated with 20 J/m^2^ UV, 250 µM H_2_O_2_, 1 mM MMS, γ-irradiation (6 Gy) or 0.1 µg/ml 8-methoxypsoralen+UVA and 1 mCi/ml or 0.5 mCi/ml [^3^H]sodium acetate were added to the culture for 1 h. Cells were then washed twice with cold phosphate-buffered saline and lysed in RIPA lysis buffer (50 mM Tris-HCl (pH 7.5), 150 mM NaCl, 1 mM EDTA, 1% Triton X-100, 1 mM NaF, 1 mM sodium orthovanadate, 10 mM sodium butyrate, and a protease inhibitor cocktail (Sigma)). The lysate was centrifuged at 14,000×*g* for 15 min at 4°C. Supernatants were incubated with a rabbit anti-WRN antibody (1∶50; Novus Biologicals) for 16 h at 4°C. Each sample was then incubated with rProtein G-Agarose beads (30 µl) at 4°C for 1 h. Immunoprecipitated proteins were resolved on a 10% SDS-PAGE gel. Gels containing [^3^H]acetate-labeled WRN were first stained with Coomassie Brilliant Blue and then enhanced by impregnating with a commercial fluorography enhancing solution (Amplify, Amersham Biosciences) for 30 min. Dried gels were subjected to autoradiography at −70°C for 5–7 days.

293T cells were transfected with either 50 nM siRNA sequence against human p300 (5′-GGACUACCCUAUCAAGUAAUU-3′, Dharmacon Research, Inc.) or 50 nM siControl Non-Targeting siRNA (Dharmacon Research, Inc.) (Negative control) with or without WRN expression plasmid (3 µg) using lipofectamine 2000 (Invitrogen, CA, USA) according to the manufacturer's instructions. Cells were harvested 24 h after transfection and analyzed by Western analysis with an anti-p300 antibody (C-20, Santa Cruz Biotechnology). After p300 was silenced by siRNA, 1 mCi/ml [^3^H]sodium acetate was added to the culture for 1 h and acetylation experiment was performed as described above.

### *In vitro* acetylation assay

Purified recombinant WRN protein or WRN fragments (1.0 µg each) were incubated with 100 ng of histone acetyltransferase enzyme p300 (Jena Bioscience) in 30 µl of HAT buffer (50 mM Tris-HCl pH 8.0, 10% glycerol (v/v), 0.1 mM EDTA, 1.0 mM DTT, 1.0 mM PMSF, 10 mM sodium butyrate), and 0.1 µCi of [^14^C]acetyl CoA (Amersham Biosciences) at 30°C for 60 min. Then, the reaction mixtures were subjected to SDS-PAGE analysis. Acetylated protein was detected by autoradiography. Gels containing [^14^C]-acetate labeled proteins were fixed with 10% glacial acetic acid and 40% methanol for 70 min and were enhanced by impregnating with a fluorography enhancing solution (Amersham Biosciences) for 30 min. Gels were then dried and autoradiography was performed at −70°C for 5–7 days.

### *In vitro* GST pull-down assay from HeLa nuclear extract

The preparation of HeLa nuclear extracts (NE) and the pull down assays with the various GST-tagged WRN fragments were done as described previously [54]. Briefly, approximately 250 µg/sample of HeLa NE was incubated for 3 h at 4°C with glutathione sepharose beads (Amersham Biosciences) saturated with GST-WRN fragments. After washing, total protein was eluted in sample buffer by boiling and analyzed by Western blotting with mouse monoclonal anti-p300 antibody (1∶1000 dilution; BD Biosciences). Equal loading of GST protein and the various GST-WRN fragments were verified by amido black staining.

### The catalytic activities of WRN

Helicase, ATPase and exonuclease activity assays were conducted as previously described [55], [56]. WRN was incubated with p300 and acetyl CoA, or p300 alone or acetyl CoA alone in HAT buffer as described above for measuring the catalytic activities of WRN. For assays with immunocomplexes [27], rabbit IgG (Santa Cruz Biotechnology) or rabbit anti-WRN (Novus Biologicals) immunoprecipitates were washed three times in lysis buffer and two times in the WRN activity reaction buffer, followed by the addition of the exonuclease substrate. Helicase and exonuclease products were run on 10% native and 14% denaturing polyacrylamide gels, respectively, and ATPase samples were analyzed on a polyethylenimine-cellulose TLC and run in 1 M formic acid-0.8 M LiCl. All the reaction products were visualized with a PhosphorImager, and quantitated by ImageQuant software (Amersham).

### Co-immunoprecipitation assay

HeLa nuclear extracts were prepared as described previously [54] and were pre-cleared with rProtein G-Agarose beads (Invitrogen). Extracts (250 µg each) were incubated with either rabbit anti-WRN antibodies (1∶50; Novus Biologicals) or 5 µg of rabbit IgG (Santa Cruz Biotechnology) as a negative control for 16 h at 4°C. Each sample was then incubated with rProtein G-Agarose beads (30 µl) at 4°C for 1 h. Bound proteins were eluted by boiling in sample buffer for 5 min and analyzed by Western analysis with mouse anti-WRN (1∶250; Transduction Laboratories) or anti-p300 (1∶1000; BD Biosciences) antibodies for 16 h at 4°C followed by chemiluminescent analysis (Pierce).

### The electrophoretic mobility shift assay (EMSA)

WRN was acetylated as described above and binding reactions (20 µl) were performed in binding buffer (40 mM Tris-HCl, pH 7.0, 1 mM EDTA, 20 mM NaCl, 8% glycerol, and 20 µg/ml bovine serum albumin) using 30 fmol labeled DNA substrate (34ForkA/B [30]), and WRN (0.1 pmol) or WRN_488–1432_ fragment (0.3 and 0.6 pmol). Protein-DNA complexes were cross-linked by adding glutaraldehyde to reactions (0.05% final concentration) after incubation for 15 min at 4°C and continued to incubation for 10 min 4°C. Samples were run on 4.5% polyacrylamide (19∶1) gel and visualized using a PhosphorImager.

### Far-Western blot

Far Western analysis was conducted as described previously [57]. The recombinant GST-tagged WRN_239–499_ and WRN_488–1432_ fragments were resolved on SDS-PAGE and blotted with 5 µg/ml p300 protein. The control blot was incubated the same amount of BSA. Western analysis was carried out by using an anti-p300 antibody (1∶1000, BD biosciences) for 16 h at 4°C followed by chemiluminescent analysis (Pierce).

### Polymerase β incorporation assay

Reactions were done as previously described [26] with some modifications. Briefly, reactions (10 µl) were performed in BER buffer (50 mM HEPES, pH 7.5, 20 mM KCl, 2 mM DTT, 10 mM MgCl_2_) and contained 4 mM ATP, 20 µM each of dATP, dGTP, dTTP, and 2.2 µM [α-^32^P]dCTP, and 1 nM pol β, increasing concentrations of acetylated (as described above) or nonacetylated WRN (6 and 12 nM). Reactions were initiated by adding 12.5 nM 34 bp duplex oligonucleotide containing one-nucleotide gap at position 16, and were incubated at 37°C for 25 min, followed by termination with equal volume of stop dye. Samples were run on 20% denaturing polyacrylamide gels and visualized using a PhosphorImager.

### Cell extracts preparation and *in vitro* single nucleotide and long patch BER assay

After wild-type and WRB KD cells were treated with 5 mM sodium butyrate (NaB) (Sigma) for 24 h at 37°C, the whole cell extracts for use *in vitro* BER assays were prepared as described previously [26]. The BER assay was initiated by the addition of treated or untreated wild-type or WRN KD whole cell extracts (5 µg) and performed as described previously [22], [26].

## Supporting Information

Figure S1 WRN interacts directly with p300. Wells were coated with p300 (3.75 nM per well). After blocking with BSA, the wells were incubated with serial dilutions of recombinant WRN (0.47, 0.94, 1.875, 3.75, 7.5, 15, 30 and 60 nM per well) in the presence of 10 µg/ml of ethidium bromide. Bound WRN was detected with rabbit anti-WRN antibodies followed by colorimetric analysis. Values represent the mean of two experiments performed in duplicate and were corrected for the background signal (WRN binding to BSA).
(0.00 MB TIF)

Figure S2 (A) Upper panel: Phosphorimages of denaturing polyacrylamide gels showing *in vitro* BER products of wild type cells. Lower panel: The average fold-increase in untreated wild type compared to NaB treated wild type cell extracts from three different experiments is plotted. (B) Upper panel: Phosphorimages of denaturing polyacrylamide gels showing *in vitro* BER products of WRN KD cells. Lower panel: The average fold-increase in untreated WRN KD compared to NaB treated WRN KD cell extracts from three different experiments is plotted.
(0.23 MB TIF)

Figure S3 Measurements of strand displacement DNA synthesis by Polβ in MMS treated or untreated WRN proficient and WRNKD cells (A) Upper panel: A schematic of the 34-bp DNA substrate containing a single nucleotide gap at position 16. Lower panel: Cell extracts were prepared from treated or untreated wild type and WRN KD cells with 1 mM MMS for 1 h. 5 µg of each cell extract used in the assay and the reactions were initiated by adding 12.5 nM one nucleotide gap substrate, and were incubated for 30 minutes at 37°C. The reaction products were run on a 20% denaturing and were visualized by a PhosphorImager. (B) Quantitation of long-patch (2–19 nt) BER intermediates in cell extracts treated with/without MMS.
(0.32 MB TIF)
