# Supplementary figures and images for: Acetylation Regulates WRN Catalytic Activities and Affects Base Excision DNA Repair
Source: PLoS One. 2008 Apr 9;3(4):e1918. doi: 10.1371/journal.pone.0001918 (PMC2276247; doi:10.1371/journal.pone.0001918)

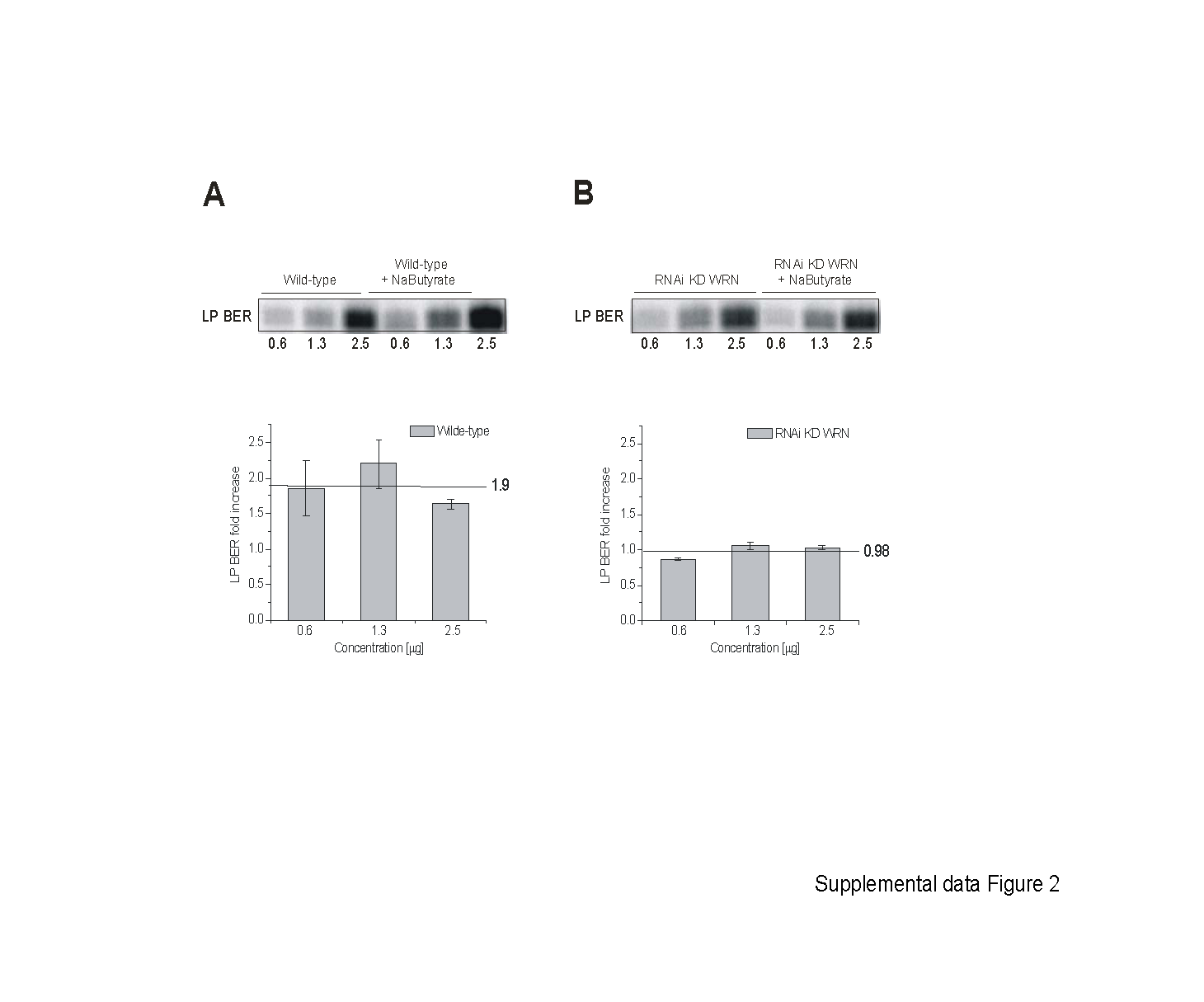

Supplement: Figure S2 — (A) Upper panel: Phosphorimages of denaturing polyacrylamide gels showing in vitro BER products of wild type cells. Lower panel: The average fold-increase in untreated wild type compared to NaB treated wild type cell extracts from three different experiments is plotted. (B) Upper panel: Phosphorimages of denaturing polyacrylamide gels showing in vitro BER products of WRN KD cells. Lower panel: The average fold-increase in untreated WRN KD compared to NaB treated WRN KD cell extracts from three different experiments is plotted. (0.23 MB TIF) [file pone.0001918.s002.tif]

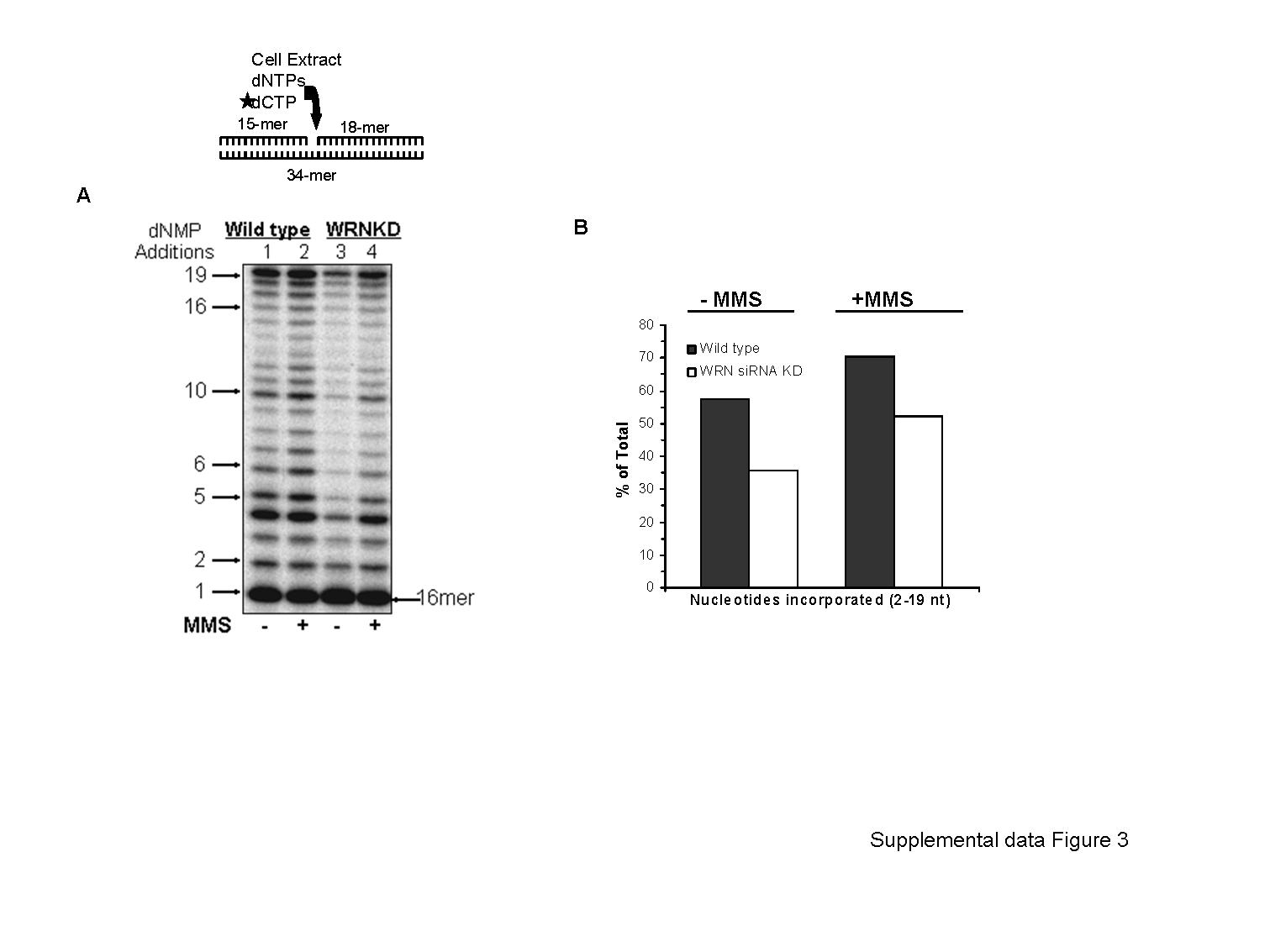

Supplement: Figure S3 — Measurements of strand displacement DNA synthesis by Polβ in MMS treated or untreated WRN proficient and WRNKD cells (A) Upper panel: A schematic of the 34-bp DNA substrate containing a single nucleotide gap at position 16. Lower panel: Cell extracts were prepared from treated or untreated wild type and WRN KD cells with 1 mM MMS for 1 h. 5 µg of each cell extract used in the assay and the reactions were initiated by adding 12.5 nM one nucleotide gap substrate, and were incubated for 30 minutes at 37°C. The reaction products were run on a 20% denaturing and were visualized by a PhosphorImager. (B) Quantitation of long-patch (2–19 nt) BER intermediates in cell extracts treated with/without MMS. (0.32 MB TIF) [file pone.0001918.s003.tif]
